# Correction: Validity and reliability of the Professionalism Assessment Scale in Turkish medical students

**DOI:** 10.1371/journal.pone.0300857

**Published:** 2024-03-14

**Authors:** Esra Çınar Tanrıverdi, Mehmet Akif Nas, Kamber Kaşali, Mehmet Emin Layık, A. M. Abd El-Aty

The fifth author’s initials appear incorrectly in the citation. The correct citation is: Tanrıverdi EÇ, Nas MA, Kaşali K, Layık ME, Abd El-Aty AM (2023) Validity and reliability of the Professionalism Assessment Scale in Turkish medical students. PLoS ONE 18(1): e0281000. https://doi.org/10.1371/journal.pone.0281000.
