# Supplementary material for: Validity and reliability of the Professionalism Assessment Scale in Turkish medical students
Source: PLoS One. 2023 Jan 26;18(1):e0281000. doi: 10.1371/journal.pone.0281000 (PMC9879428; doi:10.1371/journal.pone.0281000)
Supplement: S1 File — (DOCX) [file pone.0281000.s001.docx]

**Professionalism assessment scale for medical students**

*Please rate the extent to which you agree with the following statements which define your views on professionalism. For every item you have to choose your response on a scale from 1 (I do not agree at all) to 5 (I agree completely). Please, circle your appropriate response.*

*Please do not skip any item.*

| **1** | Hekim hasta bakarken önyargılarını bir kenara bırakmalıdır.  (When managing patients the physician should put aside his/hers prejudices.) | 1 | 2 | 3 | 4 | 5 |
| --- | --- | --- | --- | --- | --- | --- |
| **2** | Hekimin mevcut kötü ruh hali hasta bakımını etkilememelidir.  (Current bad mood of the physician should not affect the management of patients.) | 1 | 2 | 3 | 4 | 5 |
| **3** | Hekim hastalarıyla saygılı bir ilişki içinde olmalıdır.  (Physician should have a respectful relationship towards the patients.) | 1 | 2 | 3 | 4 | 5 |
| **4** | Hekim iş arkadaşlarıyla saygılı bir ilişki içinde olmalıdır.  (Physician should have a respectful relationship towards the co-workers.) | 1 | 2 | 3 | 4 | 5 |
| **5** | Hekim sürekli mesleki eğitim almaya devam etmelidir.  (Physician should constantly engage in continuous professional education.) | 1 | 2 | 3 | 4 | 5 |
| **6** | Hekim her başvuruda hastaya yardımcı olmak için elinden gelenin en iyisini yapmalıdır.  (Physician should do his/her best to help the patient in every consultation.) | 1 | 2 | 3 | 4 | 5 |
| **7** | Hekim hastayı dış görünüşüne göre yargılamamalıdır.  (Physician should not judge the patient by appearance.) | 1 | 2 | 3 | 4 | 5 |
| **8** | Hekim hastanın anlayış düzeyine uyum sağlamalıdır.  (Physician should adapt to the level of patient's understanding.) | 1 | 2 | 3 | 4 | 5 |
| **9** | Hekim hastanın istekleri için net bir sınır belirlemelidir.  (Physician should set a clear limit to which the patient can claim his/her requests.) | 1 | 2 | 3 | 4 | 5 |
| **10** | Hekim öğrenciler için iyi bir rol model olmalıdır.  (Physician should be a good role model for students.) | 1 | 2 | 3 | 4 | 5 |
| **11** | Hekim özel ve profesyonel yaşamı arasına net bir sınır koyabilmelidir.  (Physician should be able to set a clear line between private and professional life.) | 1 | 2 | 3 | 4 | 5 |
| **12** | Hekim ekibiyle profesyonel bir ilişki kurmayı hedeflemelidir.  (Physician should aspire for a professional relationship in his/her team.) | 1 | 2 | 3 | 4 | 5 |
| **13** | İyi bir hekim olmak için çok fazla klinik bilgiye sahip olmak yeterli değildir.  (A lot of clinical knowledge is not sufficient to be a good physician.) | 1 | 2 | 3 | 4 | 5 |
| **14** | Hekim-hasta iletişimi hasta yönetiminin temelidir.  (Physician-patient communication is a basis of patient management.) | 1 | 2 | 3 | 4 | 5 |
| **15** | Hekim, hastanın tıbbi olmayan sorunlarını da (kötü maddi durum, aile ilişkilerinde sorunlar vb.) anlamaya çalışmalı ve bunları hasta bakımına dâhil etmelidir.  (Physician should try to understand also patient's non-medical problems (i.e. poor financial status, family relationship problems) and include them into consultation.) | 1 | 2 | 3 | 4 | 5 |
| **16** | Her hasta bireyselleştirilmiş bir bakımı hak eder.  (Each patient deserves an individual management.) | 1 | 2 | 3 | 4 | 5 |
| **17** | Mesleki görüşünü, hastanın anlayabileceği ve kabul edebileceği şekilde hastaya sunmak hekimin görevidir.  (It is the physician's duty to present his/hers professional opinion to the patient in such a way that the patient can understand and accept it.) | 1 | 2 | 3 | 4 | 5 |
| **18** | Hekim hasta için en iyisinin ne olduğunu her zaman bilemez.  (The physician cannot always know what is best for each patient.) | 1 | 2 | 3 | 4 | 5 |
| **19** | Hastanın mahremiyetini korumak hekimin yükümlülüğüdür.  (It is the physician's obligation to protect the confidentiality of the patient.) | 1 | 2 | 3 | 4 | 5 |
| **20** | Hekim hastaya ilgi göstermelidir.  (The physician should show interest in the patient.) | 1 | 2 | 3 | 4 | 5 |
| **21** | Hastanın bilmediği bir şey varsa hekim bunu hastaya açıkça anlatmalıdır.  (The physician should tell the patient frankly if there is something he/she does not know.) | 1 | 2 | 3 | 4 | 5 |
| **22** | Hekimin hata yapabileceği kabul edilebilirdir.  (It is acceptable that the physician can make mistakes.) | 1 | 2 | 3 | 4 | 5 |
