# Supplementary material for: Validity and reliability of the Professionalism Assessment Scale in Turkish medical students
Source: PLoS One. 2023 Jan 26;18(1):e0281000. doi: 10.1371/journal.pone.0281000 (PMC9879428; doi:10.1371/journal.pone.0281000)
Supplement: Attachment — Submitted filename: Response to Reviewers.docx [file pone.0281000.s003.docx]

**PONE-D-22-21345**

**Response to Reviewers**

Dear Sudzina and reviewers,

Thank you for giving us the opportunity to submit a revised draft of the manuscript “Validity and reliability of the Professionalism Assessment Scale in Turkish medical students” for publication in the “PLOS ONE”. We want to thank you and the reviewers for their careful and positive evaluation of our manuscript. We also appreciate the time and effort the reviewers have dedicated to providing their valuable feedback and insightful comments on our paper. Below, you can find a point-by-point response to the suggestions made by the reviewers. Changes made in the manuscript are marked “red.”

Yours truly,

The corresponding author on behalf of the authors.

**Response to reviewer # 1 comments:**

|  | **Reviewer 1 Comment** | **Author response** |
| --- | --- | --- |
| 1. | Abstract: please, add the number of invited participants and the number of participants that participated in the study. | The number of students invited and participated in the study and the questionnaire response rate were added to the abstract. |
| 2. | Methods are not well structured. For example, under the Participants, you report also. Please, be consistent. | The method section has been restructured. |
| 3. | I am not sure about the sampling procedure.  1. Did you invite all students?  2. How did you invite them?  Line 117: Please, delete xxx. | The sampling procedure was explained in detail in the "Setting and participants" section.  1. All students (n=365) were invited to participate in the study. Thirty students participating in the pilot study were excluded, and the study was conducted with the remaining 335 students. Three hundred twenty-nine students volunteered to participate. The participation rate was 98%.  2. The students were informed via e-mail, and the survey link was shared in the WhatsApp group of the third graders. All of the students were included in the WhatsApp group.  XXX was written for blinding purposes. It was changed to Atatürk. |
| 4. | Results: At the beginning, please, state how many students participated in the study and what was the response rate.  Lines 236-238: you are repeating here the methods. Similarly, in some other places in the text under Results, you repeat the methods. This should be deleted. | For the retest, the questionnaire was resubmitted to the same group of students (n=335), and all were included. However, only 75 students answered the survey (added to the Settings and participants section).  The small number of students participating in the retest was stated as a limitation of the study.  Duplicates on lines 236-238 have been deleted. The findings section was reviewed, and the duplicates were deleted. |

**Response to reviewer # 2 comments**

|  | **Reviewer 2 Comment** | **Author response** |
| --- | --- | --- |
| 1. | In the introduction, it might be better to include more information about the Professionalism Assessment Scale (PAS), if it has been translated and validated into other languages, etc. | The authors could find no other version of the PAS tool.  (We also asked Prof. Dr. Klemenç, who developed the scale, but we could not find any information about its adaptation).  We added this information and some other informations to the introduction of the article. |
| 2. | In the method, the respondents were recruited from the third year. Is there any specific reason for selecting third-year students for this study? | In our education program, theoretical training and an open curriculum for professionalism start in the third year. For this reason, third-year students were chosen as the sample (added to the method section). |
| 3. | Please describe how the retest of 75 respondents were selected from the total of 329 respondents. | For the retest, the questionnaire was resubmitted to the same group of students (n=335), and all were included. However, only 75 students answered the survey (added to the Settings and participants section).  The small number of students participating in the retest was stated as a limitation of the study. |
